# Sources of social support and sexual behaviour advice for young adults in rural South Africa

**DOI:** 10.1136/bmjgh-2018-000955

**Published:** 2018-11-22

**Authors:** Guy Harling, Dumile Gumede, Maryam Shahmanesh, Deenan Pillay, Till W Bärnighausen, Frank Tanser

**Affiliations:** 1 Institute for Global Health, University College London, London, UK; 2 Africa Health Research Institute (AHRI), KwaZulu-Natal, South Africa; 3 Division of Infection and Immunity, University College London, London, UK; 4 Department of Global Health and Development, Harvard T.H. Chan School of Public Health, Boston, Massachusetts, USA; 5 Institute of Public Health, University of Heidelberg, Heidelberg, Germany; 6 School of Nursing and Public Health, University of KwaZulu-Natal, Durban, South Africa; 7 Centre for the AIDS Programme of Research in South Africa – CAPRISA, University of KwaZulu-Natal, Congella, South Africa

**Keywords:** South Africa, social support, advice, sexual behaviour, youth

## Abstract

**Introduction:**

While young people in sub-Saharan Africa (SSA) are at greatest risk of HIV acquisition, uptake of HIV prevention interventions among them has been limited. Interventions delivered through social connections have changed behaviour in many settings, but not to date in SSA. There is little systematic evidence on whom young SSA adults turn to for advice. We therefore conducted an exploratory cross-sectional study from whom young rural South Africans received support and sexual behaviour-specific advice.

**Methods:**

We asked 119 18–34  year olds in rural KwaZulu-Natal about the important people in their lives who provided emotional, informational, financial, physical, social or other support. We also asked whether they had discussed sex or HIV prevention with each contact named. We used descriptive statistics and logistic regression to analyse support and advice provision patterns.

**Results:**

Respondents named 394 important contacts, each providing a mean of 1.7 types of support. Most contacts were relatives, same-gender friends or romantic partners. Relatives provided most informational, financial and physical support; friends and partners more social support and sexual advice. Respondents reported discussing sexual matters with 60% of contacts. Sources of support changed with age, from friends and parents, towards siblings and partners.

**Discussion:**

Sexual health interventions for young adults in rural South Africa may be able to harness friend and same-generation kin social ties through which sex is already discussed, and parental ties through which other forms of support are transmitted. The gender-segregated nature of social connections may require separate interventions for men and women.

**Key questions**

**What is already known?**

- The relative importance of family and peers to young African adults for discussion of sexual behaviour is unclear.

**What are the new findings?**

- Relatives provide the majority of financial, physical and general information support to young adults in rural South Africa.
- Most young adults discuss sexual matters with others, with friends and same-generation relatives providing the majority of sexual discussion and advice.

**What do the new findings imply?**

- Sibling, friend and sexual partner relationships represent pre-existing sexual health discussion channels that might be leveraged to provide novel information and skills.
- It is likely to be harder to adapt young adults’ relationships with parents to include key sexual health messages.

## Introduction

HIV and other sexually transmitted infections (STIs) remain a significant concern in sub-Saharan Africa (SSA). This is particularly true for young people, who are at highest risk of HIV acquisition while aged under 30.^1 2^ HIV prevention efforts have long relied on behaviour change messaging aimed at limiting risky sexual behaviour.^3 4^ More recently, several efficacious biomedical prevention interventions have been developed, including universal test and treat, voluntary male medical circumcision, vaginal microbicides and pre-exposure prophylaxis.^5 6^ However, both behavioural and biomedical intervention approaches have been limited by low engagement and uptake among young people.

One way of improving intervention engagement and uptake is delivery through peers or influential others.^7 8^ This approach to improving intervention effectiveness in real-life roll-out of interventions draws on an understanding of social networks.^9^ In particular, it leverages pre-existing social capital in the form either of long-standing ties of trust (with peers) or respect (for influential others).^10^ Stable social ties are also particularly important for behaviour change that requires complex contagion (ie, multiple exposures to an idea are required before it is internalised),^11^ because they provide the opportunity for repeated exposure to new ideas that an intervention provided by individuals outside the local network may not.

Stable social ties are also more likely to reflect relationships that form part of the referent group for perceived social norms—both descriptive (ie, how important others are perceived to act) and injunctive (ie, whether important others are perceived to approve/disapprove of an action)^12 13^—which is likely to be crucial in determining one’s own behaviour. One plausible referent group for sexual behaviour is those with whom individuals talk about sexual matters. However, social capital built up through other interactions—for example, through emotional or social bonding, financial or physical help, or seeking advice—may lead individuals to treat such social contacts as important referents in general, and thus potentially influential on other matters, including sexual health.

Peer-led interventions have been used extensively for sexual health,^14^ although few explicitly consider social networks.^15^ Among those that have considered social networks, interventions led by opinion leaders or influential community members reduced risk behaviours among men-who-have-sex-with-men, male sex workers and women in the USA.^16–18^ Unfortunately, efforts to implement such models in lower-income settings have been less successful. A 2009 meta-analysis found peer-led HIV education interventions in low-income and middle-income countries sometimes changed knowledge and risk behaviours, but not biological outcomes.^19^ Approaches that nominally used social networks had no impact on risk behaviours in Zimbabwean beer halls^20^ and South African schools,^21^ probably due to not selecting peer-educators based on their social influence.

A key factor in developing peer-led interventions is therefore selecting the right individuals to diffuse new ideas or behaviours. Within SSA, constraining sexual norms, with a focus on virginity and associated respectability,^22 23^ are often key drivers of sexual behaviour.^24^ Parental discussion is typically limited by strong taboos.^25–27^ Peers are thus presumed to be the primary source of sex advice. In the context of HIV, there is particular concern that young men are hard to involve in risk-reducing activities,^28^ including testing, treatment and behaviour change.

It is not clear who influences the sexual behaviour of young people in SSA, particularly for young adults who have left the school environment. There are no quantitative data on who young Africans talk to about important matters, including advice relating to sexual behaviour and HIV-related activities. We therefore interviewed a wide range of young people aged 18–34 in a rural and small-town South African setting at the epicentre of the HIV epidemic, in order to quantify both existing sources of sexual behaviour advice and other sources of social support that might be potential sources of such advice.

## Methods

We conducted our study in November 2015 in the Somkhele demographic surveillance area (DSA) of the Africa Health Research Institute (AHRI), an ~438 km^2^ area of uMkhanyakude district, KwaZulu-Natal.^29^ All ~11 000 households in the DSA are interviewed triannually for a demographic survey. AHRI also offers annual questionnaires on household socioeconomic status and individual health, including sexual behaviours.^30^ The DSA is largely rural with one urban centre. HIV prevalence in this setting is almost 30% among adults,^31^ with a cumulative incidence for young women of 50% by age 25.^2^

We interviewed 119 adults split between one of the most densely populated periurban areas and one of the least densely populated rural areas. We further stratified our sample by respondent gender and age (18–24 and 25–34). To recruit participants, we drew up maps of each area based on a census conducted earlier in 2015 and progressed door-to-door through each area between 09.00 and 18.00 on Tuesdays to Saturdays; we intentionally included a weekend day to capture some of those who work during the week. Any age-eligible individuals present and living in each household were invited to participate until each age-gender-area stratum was roughly filled. We did not go back to households whose age-eligible members were not present. Our sample size was based on the number of respondents required to provide 80% power at α=0.05 to see a 25% difference in response rates in binary variables between strata defined by gender, geographical area and age-category at the respondent (rather than the contact) level.

Each interview used a mixture of computer-assisted personal interview (CAPI), led by the interviewer on a tablet computer, and computer-assisted self interview (CASI), in which the respondent entered their own responses on the tablet. The questionnaire was programmed and delivered using OpenDataKit (http://opendatakit.org). All interviews were conducted in isiZulu. Questions were developed in English, translated by experienced local translators and discussed within the study team, including bilingual members.

The questionnaire consisted of two components—one on social contacts, the other on sexual contacts—each broken into two sections: a name generator and name interpreters. Name generators ask respondents to list the people that fit various categories, using nicknames to uniquely identify each person. In the social contact section, we took the ‘exchange’ approach by asking respondents to list individuals who provided them with: emotional comfort; information or advice; financial support; physical assistance and household help; enjoyable socialisation; or who were otherwise important to them (exact wording in online supplementary content 1).^32^ Respondents could name the same person in multiple categories.

10.1136/bmjgh-2018-000955.supp1 Supplementary data

We chose the exchange approach, as opposed to asking about people respondents had interacted with (‘role relations’) or considered their friends, neighbours or kin (‘interactions’), because the latter are open to wide subjective interpretation. We used six name generators covering different types were preferable to a single, ‘affective’ one (eg, ‘Looking back over the last 6 months—who are the people with whom you discussed matters important to you?’ in the General Social Survey) since we were interested in a range of types social support, which these name generators explicitly capture. Our five specific name generators were chosen to map to support domains in the Arizona Social Support Interview Schedule,^33^ and thus cover a wide range of support types. We adjusted the wording of questions to fit the local context.

Name interpreter questions generate information about the relationships between respondents (‘egos’) and their contacts (‘alters’). We asked about the nature of the respondent’s relationship with each named contact, including whether they had discussed sex or prevention of HIV and other STIs (exact wording in online supplementary content 2). We also asked about respondents’ attitudes, beliefs and actions relating to HIV prevention and their perception of each contact’s attitudes, beliefs and actions. In the sexual contact section, we asked respondents about their sexual relationships with up to three people they had had sex within the past year.

The social contact name generators were collected using CAPI, as were social contact name interpreters not relating to sexual behaviour (eg, is this person a relative, where do they live). Sexual contact name generators and interpreters and social contact interpreters relating to sexual behaviour (eg, do you talk to this person about HIV prevention) were asked using CASI.

Public community information sessions were conducted before study commencement in both settings, to ensure the community was aware of the nature and research goals of the study. The research conformed to the principles embodied in the Declaration of Helsinki.

### Statistical analyses

In this analysis, we included only alters named in the social contact section, although there was overlap between alters from the sexual and social name generators. We grouped relatives into three generational categories: older, that is, parents, aunts/uncles and grandparents; same-generation, that is, siblings and cousins and younger, that is, children, nephews/nieces. We also grouped non-relatives into two categories: romantic partners and non-romantic non-relative contacts based on whether the respondent reported having ever had sex with each contact. We placed husbands/wives in the romantic partner category.

We first described the permutations and combinations of support types provided by contacts visually and using pairwise Pearson correlation coefficients. We stratified descriptive statistics of contacts by the age and gender of the respondent (women 18–24; women 25–34; men 18–24; men 25–34), looking at how the relationship and gender composition of respondents varied across these four respondent categories. We then described who provided each type of social support by relationship category, before running hierarchical logistic regression models (contacts nested within respondents) to determine how support provision varied by respondent and contact characteristics. We conducted similar analyses for exchange of advice relating to sexual behaviour. We stratified all regression models by respondent gender under the hypothesis that the social dynamics of each gender were different. Analyses were conducted in Stata release 14 and figures built in R V.3.4^34^ using UpSetR^35^ and ggplot2.^36^

## Results

### Social support

**Figure 1:**
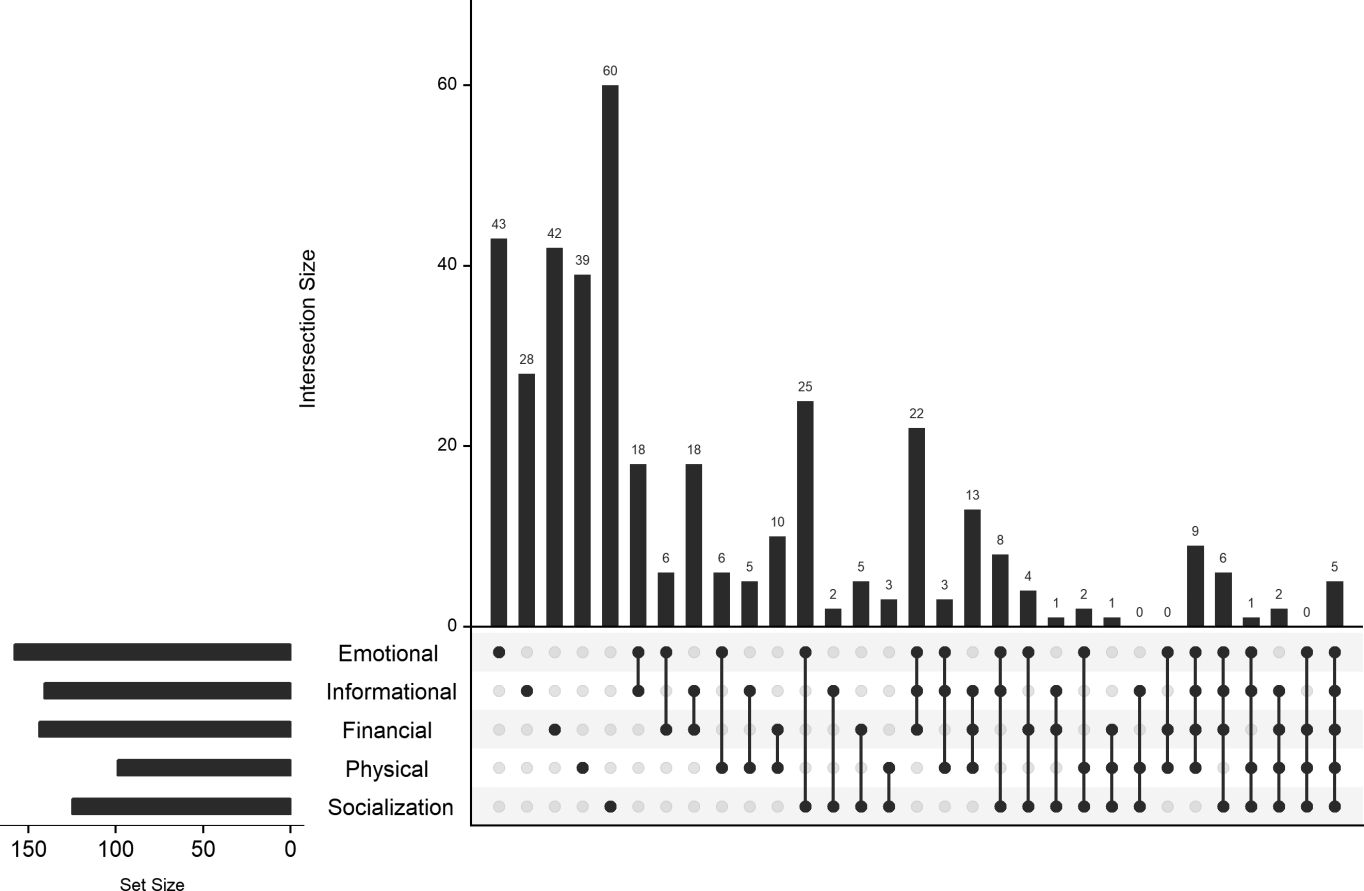
Intersection of different support types provided by social contacts. Values are for 394 contacts nested within 119 respondents. Values are the frequency of any reported support of each type (‘set size’) and the intersection of different types of support receipt (‘intersection size’). For example, 25 contacts provided both emotional and socialisation support, but no other kind.

We interviewed 119 respondents (table 1); no non-respondents were recorded. Of the 54 male respondents, 31 (57%) were aged 18–24; of the 65 female respondents, 32 (49%) were aged 18–24. The 119 respondents named 394 unique social contacts, ranging from 0 (1 respondent) to 8 each, with a median of 3 ((IQR): 2–4). Of these 394, 39 were also named as sexual contacts (44% of the total of 88 sexual contacts named). There were no significant differences in the number of social contacts named by gender or location, however, 25–34 year olds in the periurban area reported significantly fewer contacts than all other groups (mean of 2.6 vs 3.5; online supplementary content 3). Among those contacts named, the most common form of support provided was emotional, followed by informational and financial; physical support was least common (figure 1). Only 5 of the 394 named contacts (1.3%) provided all five types of support. Socialisation support was the most frequent support type provided in isolation, and this is reflected in significant negative correlations between socialisation receipt and informational (ρ=−0.22, p<0.001), financial (ρ=−0.25, p<0.001) and physical (ρ=−0.22, p<0.001) support. The most frequently paired types of support provided were emotional and socialisation; the most frequent three-clique was emotional, informational and financial. These results are reflected in significant positive associations between informational and emotional support (ρ=0.17, p<0.001) and informational and financial support (ρ=0.27, p<0.001). The only other significant pairwise correlation was between emotional and physical support (ρ=−0.16, p=0.001). Respondents reported definitely trusting the opinion of the great majority of social contacts (362/394; 91%), and over 95% of those contacts who provided emotional or informational support.

**Table 1:** Descriptive statistics for respondents and their social contacts

	Respondents		Social contacts	
	N	%	N	%
Total	119		394	
Gender				
Female	65	54.6	234	59.4
Male	54	45.4	160	40.6
Respondent and contact same-gender				
Yes			254	64.5
No			140	35.3
Age				
18–24 years	63	52.9		
25–34 years	56	47.1		
Relationship of contact to respondent*				
Grandmother/grandfather			11	2.8
Mother/father			99	25.1
Aunt/uncle			14	3.6
Husband/wife			7	1.7
Brother/sister			98	24.9
Cousin			10	2.5
Son/daughter/niece/nephew			5	1.3
Other romantic partner			47	12.0
Friend			84	21.3
Other non-relative			16	4.1
Location of respondent				
Periurban	59	49.6		
Rural	60	50.4		
Location of contact relative to respondent†				
Same household			167	42.4
Same community			120	30.5
Same district			48	12.2
Outside district			54	13.7
Contact provision of support to respondent				
Emotional			158	40.1
Informational			141	35.8
Financial			144	36.5
Physical			99	25.1
Socialisation			125	31.7
Advice flows between respondent and contact				
Ever discussed sexual behaviour			204	51.8
Ever discussed STI prevention			224	56.9
Contact ever given respondent HIV prevention advice‡			170	43.6
Contact ever given respondent partner advice‡			46	11.8

Percentages are of all social contacts for which each question was asked, ie they sum to 100% once missing data are included.

*Two contacts were unspecified relatives.

†Five contacts' locations were not specified.

‡Question not asked for four individuals who declined to answer ‘ever discussed STI prevention’ question.

**Figure 2:**
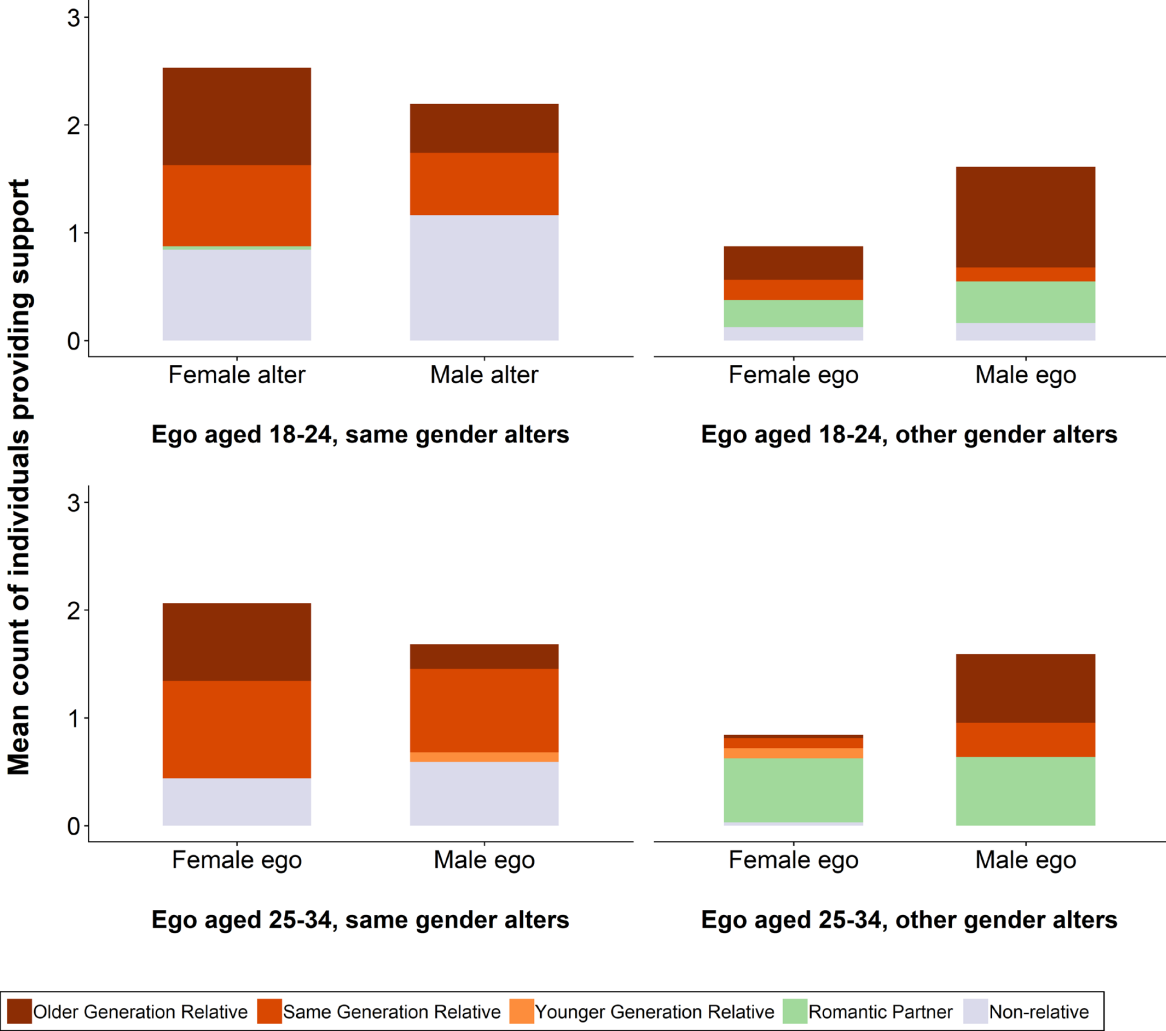
Social support receipt stratified by recipient’s age and gender and by provider’s gender and relationship to recipient. Values are for 392 contacts nested within 119 respondents. We exclude two 'unspecified relative' contacts. The left-hand side of the figure shows all same-gender social contacts; the right-hand side all other-gender social contacts. The top half of the figure shows social contacts for those aged 18–24 (63 respondents with 149 same-gender and 78 other-gender contacts); the bottom half social contacts for those aged 25–34 (55 respondents with 105 same-gender and 62 other-gender contacts).

The majority of those providing support to these young people were relatives, approximately evenly split between same-generation and older generations, with substantial minorities of non-relatives (largely friends) and romantic partners (table 1). However, these patterns vary with respondent gender and age (figure 2). First, older respondents (ie, aged 25–34 vs 18–24) report fewer non-romantic non-relatives and more romantic partners. Since almost all non-romantic non-relatives were same-gender for both men and women, the ratio of same-gender than other-gender contacts thus falls with age. Second, older respondents report receiving more support from same-generation and less support from older-generation relatives. Since everyone received substantially more support from their mothers than their fathers, and since the great majority of same-generation contacts are also same-gender, these changes lead to almost all other-gender contacts for 25–34 year olds being either mothers or sisters (for men) or romantic partners (for everyone). Notably, the 32 female respondents aged 25–34 named only 27 other-gender providers of support: 19 romantic partners, seven male relatives and one male friend.

**Figure 3:**
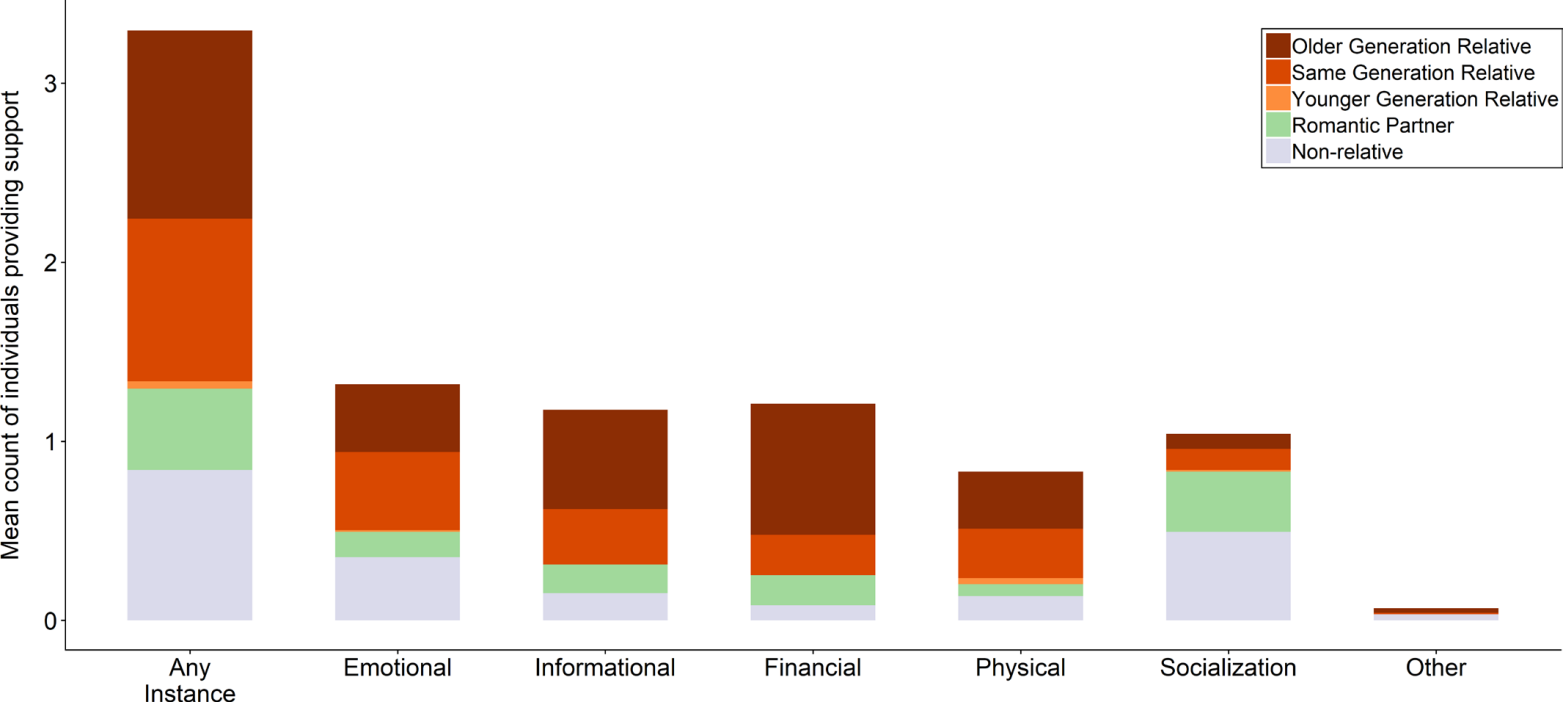
Social support receipt stratified by type of support and relationship with provider. Values are for 392 social contacts nested within 119 respondents. We exclude two *unspecified relative* contacts.

Sources of support provision varied greatly by support type (figure 3). Non-relatives were far more likely to provide socialisation and to a lesser extent emotional support than other contacts. As a corollary, older relatives were more likely to provide informational and financial support, and all relatives more likely to provide physical support, than non-relatives. In bivariate regression models, female respondents reported that their male contacts were significantly less likely to provide emotional (OR: 0.14, 95% CI: 0.06–0.31) and informational (OR: 0.47, 95% CI: 0.23 to 0.97) support, and significantly more likely to provide financial (OR: 2.31, 95% CI: 1.21 to 4.39) and physical (OR: 2.49, 95% CI: 1.24 to 5.00) support (online supplementary content 4). Male respondents reported that their female contacts were significantly more likely to provide informational (OR: 2.49, 95% CI: 1.36 to 4.56) and financial (OR: 3.95, 95% CI: 2.09 to 7.45) support, and significantly less likely to provide physical support (OR: 0.45, 95% CI: 0.23 to 0.91).

Looking specifically at parents, respondents named 77 mothers and 22 fathers as important contacts. Of those aged 18–24, 76% named their mothers and 30% their fathers; for older respondents, the proportions were 52% and 5%. Named mothers were significantly more likely to provide emotional (40% vs 14%, χ^2^=5.4, p=0.02) and informational support (68% vs 23%, χ^2^=14.1, p<0.001) compared with named fathers. The proportion of named parents reported to provide physical, financial and socialisation support were non-significantly different (physical: 29% of mothers vs 32% of fathers; financial: 78% vs 73%; socialisation: 8% vs 0%). Several of these results were confirmed in multivariable regression models (table 2, top panel).

**Table 2:** Associations between respondent and contact characteristics and provision of specific types of support

	Emotional	Informational	Financial	Physical	Socialisation
All respondents					
Older generation relative	1.00	1.00	1.00	1.00	1.00
Same-generation relative	1.17 (0.67 to 2.06)	0.44 (0.25 to 0.77)	0.17 (0.09 to 0.30)	1.11 (0.62 to 1.99)	1.35 (0.56 to 3.24)
Romantic partner	1.42 (0.63 to 3.21)	0.54 (0.25 to 1.13)	0.15 (0.07 to 0.32)	0.41 (0.16 to 1.02)	60.6 (19.4 to 188)
Non-romantic non-relative	0.97 (0.54 to 1.74)	0.18 (0.09 to 0.35)	0.06 (0.03 to 0.13)	0.44 (0.22 to 0.88)	14.0 (6.36 to 30.9)
Respondent aged 25–34 vs 18–24	1.71 (1.10 to 2.66)	1.08 (0.69 to 1.70)	0.96 (0.58 to1.59)	0.71 (0.43 to 1.18)	1.33 (0.76 to 2.34)
Other vs same-gender contact	0.31 (0.17 to 0.58)	0.80 (0.45 to 1.43)	2.63 (1.41 to 4.89)	1.12 (0.61 to 2.04)	0.37 (0.15 to 0.92)
Female vs male respondent	1.31 (0.85 to 2.03)	0.79 (0.50 to 1.25)	1.57 (0.94 to 2.62)	0.90 (0.55 to 1.47)	1.20 (0.70 to 2.07)
Female respondent					
Older generation relative	1.00	1.00	1.00	1.00	1.00
Same-generation relative	1.86 (0.86 to 4.04)	0.37 (0.17 to0.79)	0.19 (0.09 to 0.42)	0.80 (0.36 to 1.80)	1.09 (0.39 to 3.08)
Romantic partner	4.26 (0.75 to 24.4)	2.48 (0.56 to 11.0)	0.39 (0.11 to 1.34)	0.32 (0.09 to 1.15)	74.9 (11.4 to 492)
Non-romantic non-relative	0.86 (0.38 to 1.93)	0.08 (0.03 to 0.25)	0.08 (0.03 to 0.22)	0.23 (0.07 to 0.76)	10.3 (3.88 to 27.3)
Respondent aged 25–34 vs 18–24	1.32 (0.70 to 2.47)	0.86 (0.44 to 1.67)	0.68 (0.35 to 1.34)	1.31 (0.64 to 2.70)	1.45 (0.69 to 3.06)
Other vs same-gender contact	0.06 (0.01 to 0.26)	0.14 (0.04 to 0.50)	2.03 (0.78 to 5.28)	3.50 (1.38 to 8.88)	0.21 (0.04 to 1.01)
Male respondent					
Older generation relative	1.00	1.00	1.00	1.00	1.00
Same-generation relative	0.95 (0.37 to 2.42)	0.79 (0.33 to 1.91)	0.14 (0.05 to 0.36)	1.27 (0.50 to 3.27)	3.20 (0.54 to 19.1)
Romantic partner	1.52 (0.54 to 4.25)	0.37 (0.14 to 0.99)	0.06 (0.02 to 0.19)		84.9 (15.1 to 476)
Non-romantic non-relative	1.65 (0.65 to 4.23)	0.50 (0.20 to 1.28)	0.04 (0.01 to 0.15)	0.43 (0.16 to 1.16)	38.8 (7.27 to 2070)
Respondent aged 25–34 vs 18–24	2.24 (1.16 to 4.30)	1.13 (0.59 to 2.18)	1.45 (0.65 to 3.26)	0.37 (0.16 to 0.84)	1.17 (0.49 to 2.78)
Other vs same-gender contact	0.75 (0.32 to 1.76)	2.43 (1.10 to 5.36)	2.34 (0.97 to 5.65)	0.56 (0.23 to 1.33)	0.80 (0.22 to 2.90)

Separate models were run for each type of support and respondent gender shown (15 models are presented here). Values are from two-level hierarchical logistic regressions. Regressions for all respondents contained 387 contacts nested within 118 respondents. Regressions for female respondents contained 199 contacts nested within 65 respondents. Regressions for male respondents contained 188 contacts nested within 53 respondents—except for physical support where no romantic partner contacts provided such support; all such social contacts were excluded so that the model converged. All models exclude five 'younger generation relative' and two 'unspecified relative' contacts, and one respondent with no contacts. Note: the intraclass correlation coefficient for all models was approximately zero, suggesting negligible variance at the respondent level; these models are therefore almost identical to single-level models ignoring the nesting of contacts within respondents.

Models stratified by respondent gender showed that support patterns differed markedly for men and women (table 2, lower panels). Women’s primary source of emotional and informational support was romantic partners, while financial and physical support were most likely to come from older (or in the case of physical, same) generation relatives. Men’s emotional support came from a wide range of relationship types, while informational, financial and physical support were provided by older or same generation relatives. For both men and women socialisation support were overwhelmingly provided by romantic partners and non-relatives.

When we formally tested for interactions between respondent gender and level of support provided by each relationship type, men were significantly less likely to have received financial or physical support from their romantic partners than were women (table 3). Other substantial (OR >2), but non-significant, differences seen were that men received: less socialisation support from older relatives; less emotional and more socialisation support from same-generation relatives; greater emotional and socialisation support from romantic partners and more informational, physical and socialisation support from non-romantic non-relatives. There were few significant differences by respondent age: older male respondents (25–34 year olds) were more likely to receive emotional, and less likely to report physical, support than younger respondents; there were no significant age differences for women.

**Table 3:** Interaction of respondent gender and respondent–contact relationship and provision of specific types of support

	Emotional	Informational	Financial	Physical	Social
Main effect					
Older generation relative	1.00	1.00	1.00	1.00	1.00
Same-generation relative	1.73 (0.83 to 3.62)	0.40 (0.19 to 0.83)	0.19 (0.09 to 0.41)	0.85 (0.39 to 1.87)	1.11 (0.39 to 3.11)
Romantic partner	0.92 (0.29 to 2.91)	0.51 (0.18 to 1.47)	0.32 (0.11 to 0.94)	0.91 (0.30 to 2.77)	39.2 (9.49 to 162)
Non-romantic non-relative	0.93 (0.42 to 2.04)	0.10 (0.04 to 0.30)	0.08 (0.03 to 0.22)	0.22 (0.07 to 0.69)	9.91 (3.81 to 25.8)
Male vs female respondent					
Older generation relative	0.88 (0.38 to 2.04)	0.98 (0.45 to 2.12)	1.02 (0.44 to 2.36)	0.91 (0.40 to 2.10)	0.35 (0.07 to 1.83)
Same-generation relative	0.39 (0.12 to 1.25)	1.27 (0.42 to 3.80)	0.81 (0.24 to 2.73)	1.83 (0.57 to 5.84)	2.18 (0.29 to 16.3)
Romantic partner	2.43 (0.58 to 10.2)	0.99 (0.25 to 3.82)	0.20 (0.04 to 0.86)		2.75 (0.36 to 21.3)
Non-romantic non-relative	1.04 (0.32 to 3.36)	2.66 (0.68 to 10.4)	0.53 (0.11 to 2.56)	3.21 (0.74 14.0)	2.94 (0.47–18.5)
Respondent aged 25–34 vs 18–24	1.77 (1.13 to 2.77)	1.09 (0.69 to 1.71)	0.94 (0.56 to 1.55)	0.68 (0.41 to 1.14)	1.34 (0.76 to 2.35)
Other vs same-gender contact	0.30 (0.16 to 0.58)	0.87 (0.48 to 1.60)	2.28 (1.20 to 4.33)	1.18 (0.63 to 2.22)	0.43 (0.17 to 1.10)

Separate models were run for each type of support shown. Values are from two-level hierarchical logistic regressions and show main effects and gender interaction effects for each type of relative. Regressions contained 387 contacts nested within 118 respondents, except for physical support where no non-romantic non-relative female contacts provided such support to males; 26 such contacts were excluded so that the model converged. All models exclude five 'younger generation relative' and two 'unspecified relative' contacts, and one respondent with no contacts.

### Sexual health and behaviour advice

Respondents reported having ever discussed sexual matters with 60% of the contacts they named: 52% about sex in general, and 57% about how to prevent HIV and other STIs (table 1). Respondents reported having received prevention advice from 76% of the latter group and having received advice on who to consider as a sexual partner from 20%. Fifteen respondents (13%) did not report discussing sex or STI prevention with anyone, of whom 10 reported having ever had sex.

**Figure 4:**
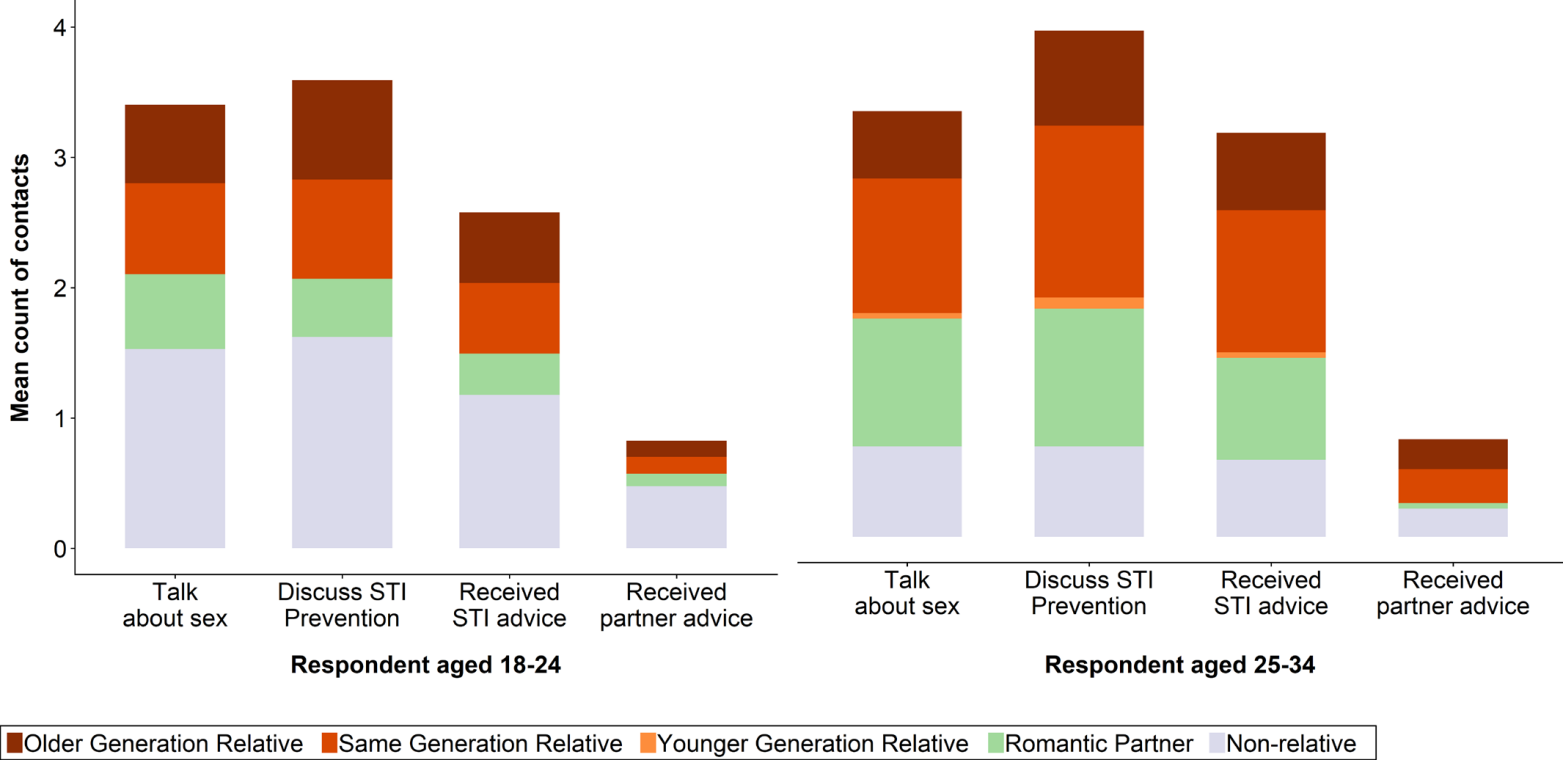
Sources of discussion and advice for sexual behaviour. Values are for 392 contacts nested within 119 respondents. We exclude two 'unspecified relative' contacts.

The types of individuals with whom respondents discussed sexual matters differed by respondent age (figure 4). Among 18–24 year olds, the largest single group of discussants was non-romantic non-relatives—particularly for partner advice. Among 25–34 year olds, same-generation relatives and romantic partners were the predominant groups, aside from almost no partner advice from romantic partners. Multivariable regression models also showed that older respondents were (non-significantly) less likely to discuss sexual matters with their non-romantic non-relatives, and more likely to do so with relatives and romantic partners, although not all these results were statistically significant (table 4). These regressions also highlight that these conversations were far less likely to take place with other-gender contacts, especially around partner advice. The substantial intraclass correlation coefficients (between 0.13 and 0.35) indicate wide variance in the level of discussion even within strata. The patterns seen in these sexual advice regressions did not vary by respondent gender.

**Table 4:** Respondent and contact characteristics predictive of discussions relating to sex

	Talked about sex	Discussed HIV prevention	Received sexually transmitted infection (STI) advice	Received partner advice
Model A				
Older generation relative	1.00	1.00	1.00	1.00
Same-generation relative	2.37 (1.24 to 4.54)	1.77 (0.96 to 3.27)	1.62 (0.83 to 3.17)	1.19 (0.40 to 3.54)
Romantic partner	115 (26.8 to 501)	20.0 (7.20 to 55.4)	10.0 (3.85 to 26.1)	4.29 (0.71 to 26.1)
Non-romantic non-relative	5.69 (2.76 to 11.7)	4.13 (2.07 to 8.23)	3.34 (1.62 to 6.87)	2.61 (0.91 to 7.43)
Respondent aged 25–34 vs 18–24	1.54 (0.84 to 2.83)	2.22 (1.25 to 3.96)	2.59 (1.36 to 4.94)	1.22 (0.48 to 3.08)
Other vs same-gender contact	0.36 (0.18 to 0.71)	0.30 (0.16 to 0.57)	0.30 (0.15 to 0.62)	0.13 (0.03 to 0.60)
Female vs male respondent	0.65 (0.35 to 1.19)	0.86 (0.49 to 1.51)	0.78 (0.41 to 1.46)	0.55 (0.22 to 1.38)
N	383	383	381	373
Intraclass correlation coefficient	0.170	0.132	0.233	0.343
Model B				
Main effect				
Older generation relative	1.00	1.00	1.00	1.00
Same-generation relative	2.21 (0.91 to 5.34)	1.54 (0.67 to 3.56)	1.30 (0.51 to 3.32)	1.37 (0.26 to 7.21)
Romantic partner	42.0 (9.08 to 194)	10.81 (3.16 to 37.0)	7.97 (2.26 to 28.1)	11.95 (1.39 to 102)
Non-romantic non-relative	6.25 (2.66 to 14.7)	5.23 (2.31 to 11.8)	3.84 (1.63 to 9.08)	4.53 (1.08 to 19.0)
Respondent aged 25–34 vs 18–24				
Older generation relative	1.37 (0.52 to 3.63)	2.14 (0.87 to 5.28)	2.44 (0.89 to 6.71)	2.82 (0.55 to 14.6)
Same-generation relative	1.21 (0.33 to 4.43)	1.33 (0.38 to 4.62)	1.52 (0.40 to 5.80)	0.68 (0.08 to 5.80)
Romantic partner		4.77 (0.61 to 37.0)	1.60 (0.30 to 8.44)	0.09 (0.00 to 1.89)
Non-romantic non-relative	0.72 (0.17 to 2.98)	0.39 (0.10 to 1.57)	0.54 (0.13 to 2.26)	0.27 (0.03 to 2.08)
Other vs same-gender contact	0.35 (0.17 to 0.69)	0.28 (0.15 to 0.54)	0.29 (0.14 to 0.60)	0.13 (0.03 to 0.60)
Female vs male respondent	0.63 (0.34 to 1.18)	0.84 (0.47 to 1.51)	0.78 (0.41 to 1.46)	0.53 (0.21 to 1.37)
N	351	383	381	373
Intraclass correlation coefficient	0.192	0.154	0.237	0.349

Values are from two-level hierarchical logistic regressions containing varying numbers of contacts nested within 118 respondents. All models exclude five 'younger generation relative' and two 'unspecified relative' contacts, four contacts who declined to answer all these questions and one respondent with no contacts. Models for receipt of STI and partner advice have additional missing observations for question-specific non-response. Each regression for Model B contains main effects and age interaction effects for each type of relative. Model B for ‘talked about sex’ dropped observations for romantic partners of 25–34 year olds since all 32 had discussed sex with the respondent.

## Discussion

In this study, we present several important findings regarding the sources of social support and sexual behaviour advice for young adults. First, relatives provide more support than non-kin, both overall and for all types of support other than socialisation; this support comes both from same-generation and older relatives. Second, and a corollary to this, friends and same-generation relatives largely provide socialisation and sexual advice, but not financial or physical support. Third, sources of advice change with age, shifting from friends and parents (especially mothers), to siblings and romantic partners. Fourth, support is primarily provided by same-gender contacts, with the notable exceptions of romantic partners and mothers (for men).

The high level of support from relatives was notable across a wide range of support types. Parents and grandparents provided more informational and financial support than any other group. This pattern of support arises in a social context of generalised poverty relative to the South African average, and extremely high youth unemployment.^37^ While such situations have been hypothesised to nudge young people, especially women, towards seeking financial support from richer and sometimes older men,^38 39^ we find that women’s older relatives had 2.5 times the odds of providing financial support compared with their romantic partners (the ratio for men was substantially higher). Our findings suggest that, in this setting, youth unemployment may have created a reliance on older kin that is more substantial than one connected to sexual favours. This financial support from older relatives may also be linked to social expectations of younger generations providing support in later years: help from later-working age individuals now may come with an implicit expectation of reciprocal support in the future.

The importance of friends and same-generation relatives for socialisation support and sexual advice is not surprising. There has been extensive research into the roles of peers, parents and media in influencing adolescent sexual behaviour in higher-income settings,^40 41^ and to a lesser extent in SSA.^42–44^ However, this work provides a first quantitative insight into the relative importance of family and peers for sexual behaviour change in South Africa, and unusually extends into early adulthood. Our evidence that sources of advice shift with age across early adulthood in this sample is novel, highlighting that although relatives provide broad informational support, friends and partners are far more involved in sexual discussions. It will be important to further explore how the content of these discussions differs by contact type and age, and to what extent different sources provide risk-reducing or risk-escalating advice. It will also be important to understand how key life events such as leaving education, moving for work and having children change both individuals social networks and the sources of influence within them.

The importance of gendered norms of behaviour is very clear in our data. Overall, the degree of gender homophily (ie, men connected to other men, women to women) is high and rises with age. Gender homophily has been seen in social networks^45^ and sexual conversation networks^46–48^ worldwide. In our data, we see stronger gender homophily in discussions about sexual behaviour than for other social support, including general sources of information. The key points of divergence from gender homophily are for general informational support (women provide more to both genders) and physical support (men provide more to both genders) and mothers provide more support to everyone than do fathers.

Our findings have several implications for the design of interventions to reduce HIV and other STI risk. First, our young adults discussed sexual matters with a majority of their close social contacts, particularly peers. These relationships with siblings, friends and sexual partners represent existing channels that can be leveraged to increase knowledge, motivation and safer sex behaviour. Given the high level of gender homophily seen in these peer relationships (aside from sexual ones), it is likely that interventions will need to ensure wide coverage of men and women separately, particularly for participants over age 25.

Second, although young adults do not much discuss sexual matters with parents in this setting, they do continue to receive other forms of parental support, including general informational support, well into adulthood. There may therefore still be an opportunity to turn these conversations towards sexual health and protection from HIV; respondents reported having discussed HIV prevention with 35 of 100 parents named as social contacts. Past parent–adolescent communication research in SSA has shown that most communication is limited and fear based,^26 27 49^ but that interventions can successfully improve levels of sexual health communication and shift content towards risk-reduction messaging.^50 51^ Work to extend this approach to young adults might be worth consideration, with the proviso that changing parent–child communication modalities once the children are themselves adults may be difficult.

Third, a minority of respondents (8%) who have had sex do not report having ever talked about sex or HIV prevention with any of their key social contacts. Such individuals may be at particular risk of poor sexual health outcomes and would be important to engage through any peer-based intervention strategy. It is also important to note that the substantial intraclass correlation coefficients seen in the sexual advice analyses implies that there was substantial heterogeneity in how respondents discussed sexual matters, and thus multiple approaches may be needed.

### Strengths and limitations

Given the strong patterns of how social support varied by respondent age and gender, and social contact gender and kinship, our work presents several testable hypotheses for future larger studies.

Our study should nevertheless be considered in the light of some potential limitations. First, we had limited power to see significant associations in the data for interactions and stratified analyses. While we saw substantial point estimates for interaction terms in table 3, in this sample, we cannot tell if these associations reflect true effects or random variation. However, there were no reported refusals to participate, reflecting the reality that data were collected by an experienced local research team at the household of respondents, and the interview process took less than 30 minutes to complete. This lack of refusal suggests that the responses collected reflected the local population present at the time interviews were conducted. Our sample is nevertheless likely to be biassed towards the 48.7% of age-eligible individuals neither in education or employment according to the 2015 annual AHRI census, who are likely to have been more often present for data collection, at least from Tuesdays to Fridays. Insofar as those in education and employment differ from their peers in terms of social and sexual relations, our findings may not generalise to the entire age-eligible population in this area.

Second, all of our data is self-reported and in this study we cannot validate respondent responses against those given by social contacts. While the topic of friendship is not typically considered a sensitive one, there may be social relationships that respondents prefer not to discuss. However, unless this social desirability bias is differential by respondent age or gender, it should not affect the key results we present here. Nevertheless, using sociocentric data methods (ie, where we can link respondents together) would allow us to validate reported relationships; this would require additional information not collected in this study, but which might be included in future work.

Third, our data are cross-sectional, which does not allow us to determine whether the named contacts actually influenced the attitudes or behaviours of respondents. Future longitudinal data would allow us to determine which types of social support provision (if any) are associated with changes in attitudes or behaviours, something that is vital if we aim to select specific social contacts to act as assistants in the delivery of HIV-related interventions.

Finally, the findings in this study are likely to be specific to rural and small-town African settings, possibly only within South Africa or even KwaZulu-Natal. Social dynamics in cities, where a greater range of social contacts are available, may be very different from those in more sparsely populated areas. Findings in this study could thus usefully be compared with future work in other South African and African settings.

## Conclusion

In this study, we showed that young adults who are members of a cohort at substantial risk of acquiring HIV and other STIs in rural and periurban South Africa receive social support from a range of different sources, and that these sources are strongly affected by gender and to a lesser extent by age. These results suggest that there may be a clear opportunity to harness peer and kin social networks to deliver effective HIV prevention interventions. However, any effort to influence the attitudes, beliefs and behaviours of these young adults via their friends and family will need to be tailored by age and gender.
