# Supplementary material for: Sources of social support and sexual behaviour advice for young adults in rural South Africa
Source: BMJ Glob Health. 2018 Nov 22;3(6):e000955. doi: 10.1136/bmjgh-2018-000955 (PMC6254751; doi:10.1136/bmjgh-2018-000955)
Supplement: Supplementary data [file bmjgh-2018-000955supp001.pdf]

Supplementary Material

Title: Sources of social support and sexual behaviour advice for young adults in rural South Africa

Supplementary Content 1. Social contact name generators use in this trial

Interview script: We would like to make a list of the people who are important to you. We will ask you several questions. For each question, there may be several people who fit the description given, or none. You can report the same person more than once if they fit several descriptions. There is no right number of answers to each question. For each person, you can give either the name you know them by, or a made-up name or a nickname if you would prefer.

N001. Who are the people with whom you could confide in or talk to about your life or your problems? For example, when you are feeling sad, anxious or upset.

N002. Who are the people who could give you information or advice in relation to an important life decision, such as a health or employment problem, or moving to another place?

N003. Who are the people you could talk to if you needed help with money, for instance borrowing money or food, or getting a job that pays?

N004. Who are the people you could ask for help with doing things? This might include tasks inside or outside your home, or borrowing tools or other small things.

N005. Who are the people you really enjoyed socializing with? This might include people who have visited you, or whom you visited, or with whom you went out somewhere?

N006. Please list anyone who is especially important to you whom you have not listed in one of the previous questions.

Supplementary Content 2. Questions about conversations relating to sexual behaviour and advice used in this trial

In this section {Nickname} refers to the name provided by the respondent for each person named in questions N001 to N006. For all questions, respondents could answer 'prefer not to respond'.

J001. Have you ever talked with {Nickname} about ways to avoid HIV or other sexually transmitted infections?

a) Yes
b) No

J002. Have you ever talked with {Nickname} about sexual matters?

a) Yes
b) No

J003. Have you ever asked {Nickname} for advice about who to have a sexual relationship with?

a) Yes
b) No

J004. Have you ever asked {Nickname} for advice about how to avoid getting HIV or other sexually transmitted infections?

a) Yes
b) No

J005. Has {Nickname} ever asked you for advice about who to have a sexual relationship with?

a) Yes
b) No

J006. Has {Nickname} ever asked you for advice about how to avoid getting HIV or other sexually transmitted infections?

a) Yes
b) No

Supplementary Content 3: Mean number of social contacts named by respondent age, gender and geographic location

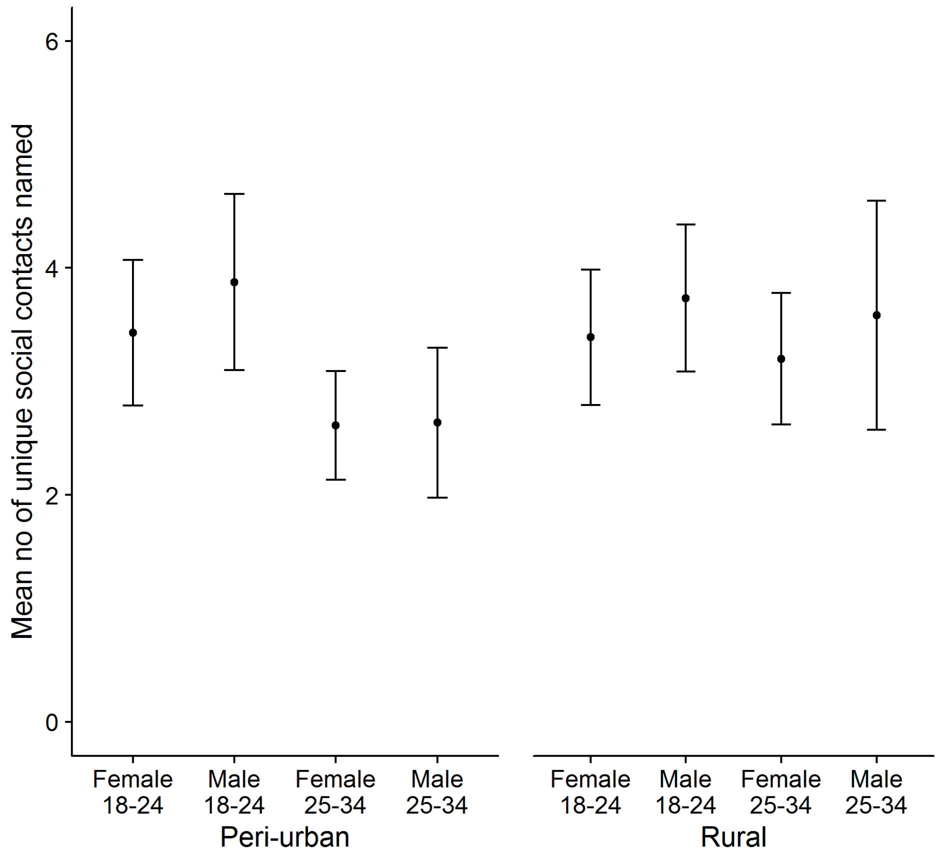

Point estimates and 95% confidence intervals for mean number of unique social contacts named by respondents.

Respondent location	Respondent age	Respondent gender	N	Mean	95%CI	Median	IQR
Peri-urban	18 to 24	Female	14	3.43	[2.79 - 4.07]	3.5	[3 - 4]
Peri-urban	18 to 24	Male	16	3.88	[3.10 - 4.65]	3.5	[3 - 4.5]
Peri-urban	25 to 34	Female	18	2.61	[2.13 - 3.09]	3	[2 - 3]
Peri-urban	25 to 34	Male	11	2.64	[1.97 - 3.30]	2	[2 - 3]
Rural	18 to 24	Female	18	3.39	[2.79 - 3.98]	3	[2 - 4]
Rural	18 to 24	Male	15	3.73	[3.09 - 4.38]	4	[3 - 5]
Rural	25 to 34	Female	15	3.20	[2.62 - 3.78]	3	[3 - 4]
Rural	25 to 34	Male	12	3.58	[2.58 - 4.59]	4	[3 - 4]

Supplementary Content 4: Associations between contact and respondent being of the same gender and support receipt

	Female respondent		Male respondent	
**Support type**	**OR**	**95% CI**	**OR**	**95% CI**
Emotional	0.14	[0.06 - 0.31]	0.75	[0.41 - 1.39]
Informational	0.47	[0.23 - 0.97]	2.49	[1.36 - 4.56]
Financial	2.31	[1.21 - 4.39]	3.95	[2.09 - 7.45]
Physical	2.49	[1.24 - 5.00]	0.45	[0.23 - 0.91]
Social	1.75	[0.91 - 3.36]	0.80	[0.43 - 1.49]

Table presents results from 10 bivariate logistic regressions stratified by respondent gender. Odds ratios and [95% confidence intervals] are for whether same vs. other gender social contacts provide support of each type. Thus, for example, female respondents' male contacts had 0.14 times the odds of providing emotional support, compared to female respondents' female contacts, while male respondents' female contacts had 0.75 times the odds of providing emotional support, compared to male respondents' male contacts.
